# The Role of MNX1–AS1 in Ovarian Cancer Resistance and Tumor Progression via RNA–RNA Interactions

**DOI:** 10.3390/ijms27083428

**Published:** 2026-04-11

**Authors:** Alvaro Gutierrez, Carolina Larronde, Salomé Silva, Constanza Castro, Rodrigo Maldonado, Daniela León, Juan Machuca, Carmen Gloria Ili, Priscilla Brebi, Kurt Buchegger, Tamara Viscarra

**Affiliations:** 1 Laboratory of Integrative Biology, Centro de Excelencia en Medicina Traslacional, Scientific and Technological Bioresource Nucleus (CEMT–BIOREN), Universidad de La Frontera, Temuco 4810296, Chile; a.gutierrez05@ufromail.cl (A.G.); c.zambrano04@ufromail.cl (C.L.); danielaines.leon@ufrontera.cl (D.L.); carmen.ili@ufrontera.cl (C.G.I.); priscilla.brebi@ufrontera.cl (P.B.); 2 Millenium Institute of Immunology and Immunotherapy, Santiago 8331150, Chile; 3 Programa de Doctorado en Ciencias, Mención Biología Celular y Molecular Aplicada, Universidad de La Frontera, Temuco 4811230, Chile; 4 Programa de Doctorado en Ciencias de Recursos Naturales, Universidad de La Frontera, Temuco 4811230, Chile; s.silva09@ufromail.cl; 5 Departamento de Ciencias Básicas, Facultad de Medicina. Universidad de La Frontera, Temuco 4810296, Chile; c.castro25@ufromail.cl; 6 Facultad de Ciencias, Universidad San Sebastián, Valdivia 5110246, Chile; rodrigo.maldonado@uss.cl; 7 BMRC, Biomedical Research Consortium, Santiago 8331150, Chile; 8 Programa de Doctorado En Ciencias Biomédicas, Facultad Ciencias de la Salud, UniversidadAutónoma de Chile, Temuco 4780000, Chile.; juan.machuca@cloud.uautonoma.cl; 9 Facultad de Ciencias de la Salud, Instituto de Ciencias Biomédicas, Universidad Autónoma de Chile, Temuco 4780000, Chile

**Keywords:** ovarian cancer, lncRNA, MNX1–AS1, chemoresistance, epithelial–mesenchymal transition

## Abstract

Ovarian cancer (OC) remains one of the deadliest gynecological malignancies, largely due to late diagnosis and the emergence of resistance to platinum–based chemotherapy. Long non–coding RNAs (lncRNAs) have recently emerged as key regulators of tumor progression and therapeutic adaptation. In this study, we performed integrative transcriptomic profiling of patient–derived TCGA ovarian tumor samples and carboplatin–resistant A2780 (CBDCA–R–A2780) cells to identify lncRNAs whose dysregulation overlaps between a cell–line resistance model and patient tumors. Our analyses revealed extensive transcriptional remodeling across both datasets, with *MNX1*–*AS1* consistently emerging as a strongly deregulated transcript. Differential expression analysis showed robust upregulation of *MNX1*–*AS1* in resistant cells and tumor tissues, accompanied by correlations with epithelial–mesenchymal transition (EMT)–related transcription factors such as *FOXA1* and *SNAI2* and inverse associations with epithelial markers including *CDH1*. Computational predictions using RIblast identified specific *MNX1*–*AS1* binding regions with candidate miRNAs and mRNAs, prioritizing EMT–related transcripts (e.g., *SNAI2*, *FOXA1*, *ZEB1*) with favorable hybridization energies for future validation. Additional prioritized interactors included genes linked to stress response (*IER2*, *FOSB*) and invasion (*MMP11*, *MMP1*). Because A2780 has been discussed as an endometrioid–like/non–serous ovarian cancer model, mechanistic inferences primarily apply to this in vitro context, while TCGA analyses provide associative support rather than mechanistic validation. Collectively, these findings highlight *MNX1*–*AS1* as a candidate regulator associated with transcriptional reprogramming in OC and a promising prognostic biomarker warranting further functional testing.

## 1. Introduction

Ovarian cancer persists as one of the most lethal gynecological malignancies worldwide, claiming approximately 150,000 lives annually [1]. Despite considerable advances in therapeutic approaches and surgical techniques, the five–year overall survival rate remains discouragingly low at 30–40%, primarily due to late–stage diagnosis and the development of chemoresistance [2]. Within the spectrum of ovarian malignancies, epithelial ovarian cancer (EOC) accounts for approximately 90% of cases, with high–grade serous ovarian carcinoma (HGSOC) representing the most aggressive and prevalent form [3]. Importantly, commonly used ovarian cancer–cell–line models (including A2780) may not fully recapitulate HGSOC biology; this limitation is considered throughout our interpretation of TCGA associations and in vitro findings.

The clinical challenges of ovarian cancer present formidable obstacles to improving patient outcomes. Current epidemiological data illustrates the magnitude of this health crisis, with China alone reporting approximately 52,100 new cases and 22,500 deaths annually [4]. Similarly concerning statistics emerge from the United States, where annual projections indicate 22,440 new cases and 14,080 deaths [1]. The disease disproportionately affects postmenopausal women, with peak diagnosis occurring between ages 55 and 64. Several risk factors have been well–documented, including *BRCA1* and *BRCA2* genetic mutations, family history of ovarian or breast cancer, nulliparity, early menarche, and late menopause [5].

A critical challenge in disease management lies in the fact that 70–75% of patients receive their diagnosis at advanced stages, after metastasis has occurred [6]. Current treatment protocols typically combine aggressive cytoreductive surgery with platinum–based chemotherapy, usually pairing carboplatin with paclitaxel [7]. Although initial response rates reach 60–80%, most patients develop resistance to platinum–based therapy within six months, underscoring the urgent need for novel therapeutic strategies [8].

Recent technological advances, particularly in high–resolution microarray and massively parallel sequencing, have transformed our understanding of genome regulation and cancer biology [9]. These developments have revealed that over 98% of the human genome transcribes into non–protein–coding RNA molecules [10]. Among these, long non–coding RNAs (lncRNAs), defined as transcripts exceeding 200 nucleotides, have emerged as crucial regulators of cellular processes [11].

The significance of lncRNAs in cancer biology has grown exponentially, as evidenced by the Encyclopedia of DNA Elements (GENCODE) database, which documents an increase in identified lncRNA genes from 6496 in 2009 to 15,941 in 2015 [12]. These molecules demonstrate remarkable regulatory versatility, interacting with DNA, chromatin, proteins, and various RNA species to modulate gene expression and cellular function [12,13].

LncRNAs operate through complex and diverse mechanisms, functioning as chromatin modifiers by recruiting and directing chromatin–modifying complexes to specific genomic regions, thereby influencing histone modifications and DNA methylation patterns [14]. At the transcriptional level, they serve as scaffolds for transcription factor assembly and regulate enhancer–promoter interactions [15]. Post–transcriptionally, they influence mRNA splicing, stability, and translation [16].

In ovarian cancer specifically, dysregulated lncRNAs play crucial roles in disease pathogenesis [17]. These molecules influence multiple aspects of tumor biology, including cell proliferation, survival, metastasis, and drug resistance. Notable examples include *HOTAIR*, *MALAT1*, and *H19*, which promote cell proliferation and inhibit apoptosis through complex regulatory networks [18,19]. For instance, *HOTAIR* enhances tumor growth by modulating the PI3K/AKT signaling cascade, while *MALAT1* influences cell survival through multiple pathways [20].

Of particular significance is the role of lncRNAs in chemoresistance development, a critical barrier to successful treatment. Research has demonstrated their involvement in regulating drug efflux pathways, DNA damage repair processes, and apoptotic responses [21]. These molecules also influence the tumor microenvironment, affecting both immune response and angiogenesis, thereby contributing to treatment resistance and disease progression [22].

Recent studies have highlighted the crucial role of lncRNAs in metabolic reprogramming of cancer cells. These molecules regulate various metabolic pathways, including glucose metabolism, fatty acid synthesis, and oxidative phosphorylation [23]. Such metabolic alterations prove fundamental to cancer cell survival and proliferation, particularly under chemotherapy–induced stress conditions. Furthermore, certain lncRNAs can influence key metabolic enzyme activity, suggesting potential therapeutic opportunities through metabolic targeting [24].

The therapeutic potential of targeting lncRNAs has generated considerable interest within the research community. Various approaches under investigation include antisense oligonucleotides, small–molecule inhibitors, and CRISPR–based technologies [25]. While early studies suggest that modulating specific lncRNAs might enhance chemotherapy sensitivity and reduce metastatic potential, significant challenges remain in developing effective delivery systems and minimizing off–target effects [26].

In this context, the long non–coding RNA *MNX1*–*AS1* has emerged as a key player in various oncological settings. Its overexpression has been reported in gastric, lung, breast, and ovarian cancers, correlating with adverse clinical parameters such as larger tumor size, lymph node metastasis, and poor prognosis [27]. At the molecular level, *MNX1*–*AS1* exerts oncogenic functions through multiple mechanisms: it can act as a competing endogenous RNA (ceRNA), regulate transcription factors, associate with RNA–binding proteins, and modulate critical signaling pathways [27]. Notably, in breast cancer, *MNX1*–*AS1* activates the PI3K/AKT pathway by enhancing *ITGA6* expression, thereby promoting paclitaxel resistance. In ovarian cancer, emerging evidence, including our analyses, demonstrates that *MNX1*–*AS1* is consistently upregulated in carboplatin–resistant models and associates with EMT–related genes, supporting its role as a potential prognostic biomarker and therapeutic target [28].

The objective of this research is to identify potential lncRNAs associated with therapeutic failure and chemoresistance in ovarian cancer. Furthermore, this study aims to elucidate the possible interactions these candidates may have with other molecules influencing these phenomena, as well as the molecular structure that the candidate lncRNAs may possess. This knowledge is fundamental for the development of novel diagnostic and therapeutic strategies to overcome treatment resistance and improve the prognosis of ovarian cancer patients.

## 2. Results

### 2.1. Identification of Differentially Expressed Genes and LncRNAs in Ovarian Cancer and Platinum Resistance

#### 2.1.1. Transcriptomic Landscape in Ovarian Cancer Patients (TCGA–OV)

To identify key molecular players in ovarian cancer (OC), we first analyzed public data from the TCGA–OV cohort compared to normal ovarian tissue from GTEx. Our analysis revealed that 312 genes were significantly upregulated and 227 genes were significantly downregulated (FDR–adjusted *p* < 0.05). Among the most significantly upregulated genes in tumor samples, *MMP11*, *S100P*, and *KRT17* showed the greatest increases in expression. Additional upregulated genes included *CEACAM5* and *SPINK1*. Several of these transcripts are involved in extracellular matrix remodeling, epithelial differentiation, and tumor progression, suggesting activation of oncogenic transcriptional programs. In contrast, genes such as *ADH1B*, *PDZK1IP1*, and *FMO5* were significantly downregulated in tumor samples relative to normal tissues. These genes are primarily associated with metabolic homeostasis and epithelial function, indicating a disruption of normal–tissue–specific transcriptional programs during tumorigenesis. The observed expression patterns reflect the transcriptional reprogramming characteristic of tumor tissues, marked by activation of proliferative and invasive pathways and suppression of normal epithelial and metabolic gene networks. Full gene lists, including fold changes and *p*-values, are provided in Table S1—Sheet DEG—TCGAvGTEx.

Regarding the non–coding transcriptome, 6867 lncRNA transcripts were quantified in the TCGA cohort. Of these, 4419 were significantly upregulated and 2448 were significantly downregulated. Ranking by log_2_ fold change magnitude and statistical significance identified the most prominent alterations. Among the most strongly upregulated lncRNAs, *LINC00221* and *LINC00460* displayed consistent transcriptional induction in tumor tissues. Additional examples included *CDKN2B*–*AS1* (ANRIL), a transcript linked to regulation of the *INK4* locus. Conversely, several well–characterized lncRNAs were significantly downregulated. These included *MEG3*, a tumor suppressor; *HOTAIR*, implicated in chromatin remodeling; and *ZEB2*–*AS1*, associated with epithelial–mesenchymal transition (EMT).

#### 2.1.2. Differential Expression in Carboplatin–Resistant Models (CBDCA–R–A2780)

To complement the clinical data, we analyzed the transcriptomic profile of parental A2780 cells versus carboplatin–resistant (CBDCA–R–A2780) lines. This comparative analysis identified a substantial number of differentially expressed genes (DEGs), ranked by fold change magnitude and FDR–adjusted *p*-values (*p* < 0.05). Overall, the model identified 126 significantly upregulated and 89 downregulated genes, with full details provided in Table S1—Sheet–resistant cell lines.

The resistant phenotype was characterized by a robust induction of stress response and cellular adaptation pathways. Among the most significantly upregulated genes, *MT1X*, *MT2A*, and *S100A2* exhibited the highest increases in expression, alongside notable upregulation of *FOSB* and IER2. This transcriptional reprogramming suggests a clear shift toward stress resilience and adaptive survival. Simultaneously, a marked downregulation was observed in genes maintaining epithelial integrity and cell adhesion, most notably *KRT8*, *EPCAM*, and *SPP1*. These shifts reflect the phenotypic changes and reduced epithelial characteristics typically associated with acquired resistance.

In parallel, evaluation of the lncRNA transcriptome revealed a specific signature of the resistant state. Ranking by fold change and significance revealed substantial deregulation, with *LINC02258* and *LSAMP*–*AS1* emerging as the most upregulated candidates. Other transcripts showing marked induction included *NIHCOLE*, *GPC5*–*AS2*, *GPC5*–*IT1*, *LINC02336*, *LINC02404*, and *MPPED2*–*AS1*. On the other hand, a distinct group of lncRNAs exhibited significant downregulation. The most striking decreases were observed for *PDGFD*–*AS1* (*PDGFDDN*) and *LINC02160*, followed by the consistent repression of *LINC01515*, *DLGAP1*–*AS5*, *LNCAROD*, *RMDN2*–*AS1*, and *OTX2*–*AS1*. Notably, *MNX1*–*AS1* was also identified as significantly upregulated (log2FC = 4.68; *p* = 3.28 × 10^−9^), underscoring its relevance within the molecular landscape of CBDCA–resistant A2780 cells.

#### 2.1.3. Intersection of Clinical and Experimental Data: MNX1–AS1 Shows Associative Concordance Across Datasets

**Figure 1:**
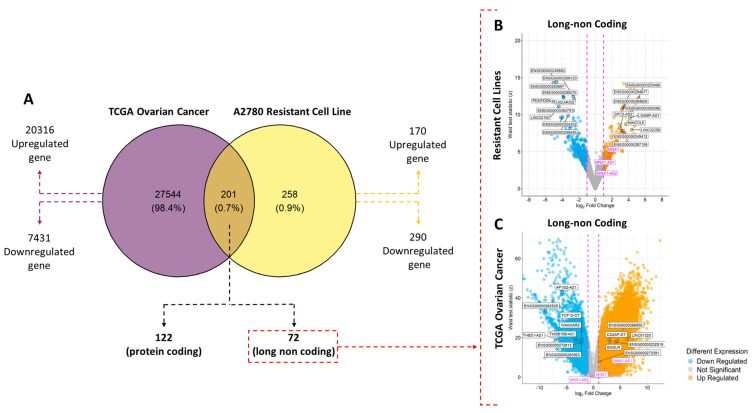
Differentially expressed genes in TCGA ovarian tumor tissue (predominantly HGSOC) and the A2780 carboplatin–resistant cell–line model. (**A**) Venn diagram represents the comparison of gene expression among 27,745 transcripts in ovarian tumor samples and 459 transcripts in the A2780 carboplatin resistance model. The intersection shows 201 transcripts with overlapping regulation between both datasets, of which 72 correspond to lncRNAs. (**B**) Volcano plot showing the magnitude of change (Log2 fold change) and statistical significance (Wald test score and *p*-values) of genes in the carboplatin–resistant A2780 model. (**C**) Volcano plot for TCGA tumor samples, highlighting the lncRNAs deregulated in the tumor context. The lncRNA *MNX1*–*AS1* (magenta in plots B and C) is overexpressed in both datasets. Full lists of deregulated lncRNAs are provided in the Supplementary Material.

Comprehensive transcriptomic profiling of RNA–seq data from patient–derived TCGA tumor samples and carboplatin–resistant cell lines (CC) derived in vitro uncovered extensive transcriptional remodeling in both datasets. Utilizing a significance threshold of log2FC > 1 and *p* value < 0.05, Venn diagrams and volcano plots (Figure 1A) were employed to delineate the unique and shared molecular signatures of these systems.

Most differentially expressed genes (DEGs) were unique to each biological system, reflecting the broad transcriptional heterogeneity of patient tumors compared to the in vitro model. The intersection of both datasets revealed 201 transcripts with convergent regulation, of which 72 correspond to lncRNAs. Comparative analysis demonstrated that resistant cells exhibited a marked upregulation of programs associated with proliferation, survival, and epithelial–mesenchymal transition (EMT). In contrast, the TCGA samples exhibited greater complexity, incorporating both tumor–intrinsic properties and microenvironmental influences.

Despite contextual differences between patient tumors and in vitro models, *MNX1*–*AS1* was upregulated in both datasets, supporting it as a recurrently deregulated lncRNA across a cell–line resistance model and patient tumors (see Table S1, Figure 1B,C). Within the *MNX1* genomic locus, *MNX1*–*AS1* was the sole transcript consistently overexpressed in both the TCGA vs. GTEx comparison and in resistant A2780 cells. *MNX1*–*AS1* emerges as the lead candidate from this overlap, prioritizing it for deeper mechanistic interrogation and motivating cross–model comparisons that link A2780 resistance–associated transcriptional shifts to HGSOC tumor transcriptomic states to delineate shared versus histology–specific resistance programs.

### 2.2. Spearman’s Correlation Related with MNX1–AS1 Analysis

**Figure 2:**
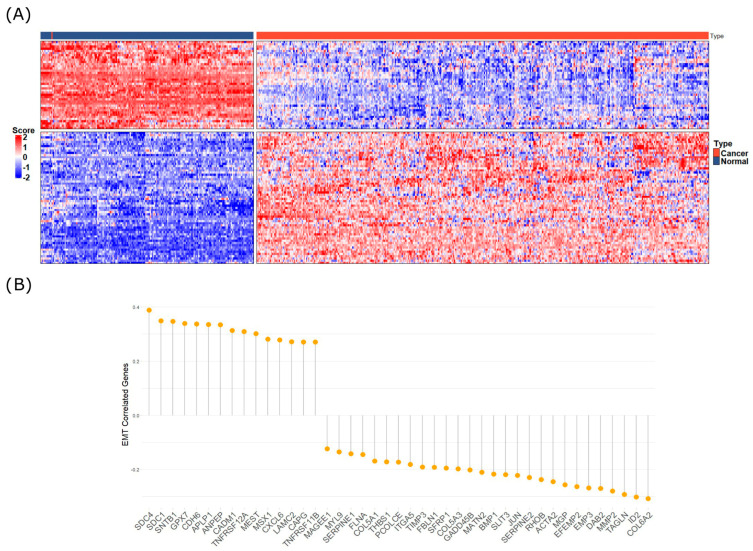
Spearman’s correlation analysis of MNX1–AS1 expression and ovarian cancer transcripts. (**A**) Heatmap of top 100 transcripts significantly correlated with MNX1–AS1 expression; for more information of each gene, see Table S2. (**B**) Deregulated and positively and negatively correlated genes and genes belonging to the EMT metabolic pathway.

Spearman’s correlation analysis was performed to identify genes transcriptionally associated with *MNX1*–*AS1* expression, see Figure 2. Genes were ranked based on the strength of correlation (Spearman’s ρ) and statistical significance (*p*-value). Among the most strongly positively correlated genes, *FOXA1*, *HOXC6*, and *SLC39A1* demonstrated the highest correlation coefficients. Additional positively correlated transcripts included *CDH17* and *SNAI2*. These results indicate a strong transcriptional association between *MNX1*–*AS1* expression and genes involved in epithelial–mesenchymal transition (EMT), transcriptional regulation, and metal ion transport. Conversely, negatively correlated genes included *CLDN1*, *CDH1*, and *TJP1*. These genes are typically associated with maintenance of epithelial integrity, suggesting an inverse relationship between *MNX1*–*AS1* upregulation and epithelial phenotype markers. Overall, 90 genes exhibited strong positive correlations, and 54 genes showed negative correlations, suggesting that *MNX1*–*AS1* expression is tightly linked to a transcriptional program favoring mesenchymal features and the suppression of epithelial identity. While these patterns align with those observed in resistance and tumor progression models (see Table S2 for full coefficients and *p*-values), these RNA–RNA interactions should be viewed as a robust framework for future functional validation rather than established biological facts.

### 2.3. Evaluation of the Interaction Between MNX1–AS1 and miRNA/mRNA

After evaluating the interaction probabilities of a total of 585 miRNAs (NCBI database) and 5656 mRNAs (NCBI database) with the accessible region of *MNX1*–*AS1* (992 bp), three predicted miRNA interaction sites were identified. These sites corresponded to nucleotide bases 62–68 bp (region A), 647–668 bp (region B), and 945–954 bp (region C) of *MNX1*–*AS1*. Region A exhibited 378 potential interactions involving 131 miRNAs. Region B showed 698 interaction probabilities across 195 miRNAs, while region C demonstrated 660 interaction probabilities involving 148 miRNAs.

**Figure 3:**
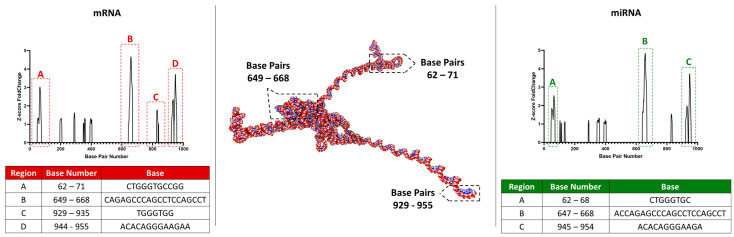
Prediction of mRNA and miRNA interaction sites with MNX1–AS1. The central structure illustrates a representation of the MNX1–AS1 secondary structure. Four key regions were identified for mRNA binding (A, B, C, D) and three for miRNA binding (A, B, C). Regions A, B and C, are shared as potential interaction points between both RNA classes. The D site is unique to mRNA interaction. The bottom tables (red (Left): mRNA; green (right): miRNA) detail the exact location (base pair number) and base sequence for each predicted interaction region.

Regarding the predicted binding sites between mRNAs and the accessible region of *MNX1*–*AS1*, four potential interaction regions were identified. Three of these regions overlapped with those predicted for miRNAs, corresponding to nucleotide bases 62–71 (region A), 649–668 (region B), and 944–955 (region D). Region C (nucleotide bases 929–935 bp) exhibited an interaction site exclusive to mRNAs, which was not shared with the miRNA–predicted binding sites, see Figure 3.

To investigate potential post–transcriptional regulatory mechanisms mediated by *MNX1*–*AS1*, predicted RNA–RNA interactions obtained from RIblast analysis were integrated with transcriptomic profiles of upregulated genes from CBDCA–resistant A2780 cells and TCGA tumor samples. This comprehensive integration allowed for the identification of transcripts that were both transcriptionally activated and predicted to physically interact with *MNX1*–*AS1*, providing insights into candidate regulatory axes in resistance and tumor progression contexts, see Figure 3.

In CBDCA–resistant A2780 cells, 42 upregulated genes were found to overlap with RIblast–predicted *MNX1*–*AS1* interactors. The predicted hybridization energies for these interactions ranged from −31.2 kcal/mol to −45.7 kcal/mol, with a median value of −36.5 kcal/mol, indicating a strong likelihood of stable RNA–RNA binding. Notable examples included *SNAI2* (log2FC = 3.87, interaction energy = −42.1 kcal/mol) and *FOXA1* (log2FC = 2.85, interaction energy = −38.3 kcal/mol). Additional overlapping genes included *SLC39A1* (log2FC = 1.92, *p* = 8.2 × 10^−4^, interaction energy = −34.6 kcal/mol) and *HMGA2* (log2FC = 2.28, interaction energy = −37.2 kcal/mol), both previously associated with processes such as stemness and adaptive stress response.

In the TCGA cohort, 56 upregulated genes were identified as predicted *MNX1*–*AS1* interactors based on RIblast analysis. The predicted hybridization energies for these genes ranged from −31.5 kcal/mol to −47.9 kcal/mol, with a median value of −37.1 kcal/mol. Genes such as *MMP11* (log2FC = 7.85, interaction energy = −44.6 kcal/mol) or *S100P* (log2FC = 6.92, interaction energy = −42.8 kcal/mol) demonstrated both robust upregulation and highly favorable binding predictions with *MNX1*–*AS1*. Due to the large number of values and genes, please see S3.

Comparison across models revealed that several key transcripts, including *SNAI2*, *FOXA1*, and *ZEB1*, were commonly upregulated and predicted to interact with *MNX1*–*AS1* in both resistant cells and TCGA tumors. These genes exhibited highly stable hybridization energies (all <−38 kcal/mol) and consistent positive fold changes, supporting a candidate interaction overlap that warrants experimental validation in appropriate subtype–specific models.

Functional annotation of the intersecting gene sets revealed enrichment for processes related to epithelial–mesenchymal transition (EMT), extracellular matrix remodeling, cell migration, and transcriptional regulation. EMT–related genes constituted approximately 25% of the intersecting transcripts, with many exhibiting interaction energies below −40 kcal/mol, reinforcing the hypothesis that *MNX1*–*AS1* may contribute to the regulation of mesenchymal programs through direct RNA–RNA contacts. Further stratification showed that the distribution of predicted interaction energies was similar between the two models. In CBDCA–resistant cells, 78.6% of the overlapping genes exhibited interaction energies lower than −35 kcal/mol, while in TCGA samples, 82.1% of the genes fell into this highly stable interaction range. This observation supports the robustness of the predicted RNA–RNA binding across independent biological systems.

Among genes unique to resistant cells, transcripts such as *IER2* (log2FC = 4.39, interaction energy = −35.4 kcal/mol) and *FOSB* (log2FC = 4.73, interaction energy = −33.8 kcal/mol) were notable for their roles in stress response and immediate–early gene activation. Conversely, unique interactors identified in TCGA included *MMP1* (log2FC = 6.28, interaction energy = −45.2 kcal/mol) and *SERPINB5* (log2FC = 5.87, interaction energy = −41.3 kcal/mol), both associated with invasion and metastatic dissemination.

Taken together, these findings demonstrate a significant convergence between RIblast–predicted RNA–RNA interactions and transcriptionally activated genes in resistance and tumorigenesis. The strong predicted binding affinities, coupled with substantial upregulation, highlight a subset of *MNX1*–*AS1*–associated genes that may represent key components of resistance–related molecular programs. These results underscore the potential importance of direct RNA–RNA interactions in modulating gene expression landscapes in both experimental and clinical settings.

Full details of the overlapping gene lists, including predicted hybridization energies, are provided in Table S3.

## 3. Discussion

The clinical significance of our findings is supported by RNA–seq analysis of patient–derived TCGA tumor samples. This cohort is enriched for high–grade serous ovarian cancer (HGSOC), the most prevalent epithelial ovarian cancer subtype, and reveals extensive transcriptional remodeling. In these patient tissues, we observed significant upregulation of oncogenic genes, such as *MMP11* and *S100P*, and downregulation of genes involved in metabolic homeostasis, such as *ADH1B*. These signatures reflect both intrinsic tumor properties and microenvironmental influences. Clinically, high *MNX1*–*AS1* expression has been reported to correlate with advanced FIGO stage, higher tumor grade, distant metastasis, and poorer overall survival (OS) and progression–free survival (PFS) [29,30], supporting *MNX1*–*AS1* as a candidate prognostic biomarker in epithelial ovarian cancer (EOC).

Model and subtype context are important considerations for interpreting these findings. While TCGA–OV is enriched for HGSOC, our mechanistic insights are derived from the A2780 carboplatin–resistance model, which has been discussed in the literature as more aligned with endometrioid–like/non–serous ovarian cancer contexts. Importantly, the concordant signal observed in TCGA supports the broader relevance of *MNX1*–*AS1* and motivates systematic, program–level and functional validation in established HGSOC models to define the extent of shared resistance biology.

Some transcriptional features observed in TCGA tumors are directionally consistent with those in our carboplatin–resistant model (CBDCA–R–A2780). In resistant cell lines, genes associated with stress response and cellular adaptation, such as *MT1X*, *MT2A*, and *S100A2*, were significantly upregulated, whereas epithelial markers such as *KRT8* and *EPCAM* were downregulated. Additionally, we identified a cohort of lncRNAs exhibiting strong repression in this model, most notably *PDGFD*–*AS1* (*PDGFDDN*) and *LINC02160*, followed by the consistent downregulation of *LINC01515*, *DLGAP1*–*AS5*, *LNCAROD*, *RMDN2*–*AS1*, and *OTX2*–*AS1*. However, similarities at the level of selected genes do not imply shared global transcriptional programs or conserved resistance mechanisms across histologies, and TCGA concordance should be interpreted as associative.

Despite heterogeneity between patient tumors and in vitro models, *MNX1*–*AS1* was among the lncRNAs upregulated in both datasets. Within the *MNX1* genomic locus, *MNX1*–*AS1* was the sole transcript consistently deregulated, which highlights it as a focused candidate for follow–up. Mechanistically, *MNX1*–*AS1* has been reported to act through competing endogenous RNA (ceRNA) networks, sequestering miRNAs to de–repress target genes. Established examples include the *MNX1–AS1/miR–744–5p/SOX12* axis, which promotes proliferation and migration, and the *MNX1–AS1/hsa–miR–4697–3p/HOXB13* axis, which has been linked to carboplatin sensitivity [30]. Nonetheless, whether analogous regulatory dependencies operate in HGSOC requires direct validation in subtype–appropriate models.

Our RIblast interaction analysis further refines this understanding, identifying three primary miRNA interaction sites (62–68 bp, 647–668 bp, and 945–954 bp) and four mRNA binding regions. Integrating these predictions with our transcriptomic data revealed that key EMT–related genes, including *SNAI2*, *FOXA1*, and *ZEB1*, are predicted to form stable interactions with *MNX1*–*AS1* (e.g., *SNAI2* at −42.1 kcal/mol; *FOXA1* at −38.3 kcal/mol). Spearman’s correlation analysis solidified this linkage, showing that *MNX1*–*AS1* expression is strongly positively correlated with mesenchymal transcription factors (*FOXA1*, *SNAI2*) and negatively correlated with epithelial integrity markers (*CLDN1*, *CDH1*), indicating a program favoring mesenchymal features.

In summary, the overlap between the A2780 resistance model and TCGA tumor data supports *MNX1*–*AS1* as a prioritized lncRNA associated with resistant–like transcriptional features, but it should be interpreted as associative rather than evidence of conserved resistance biology across histologies. While our results are currently based on correlative analyses and in silico interaction predictions, they provide a testable molecular framework that can guide follow–up experiments. In particular, RIblast–predicted RNA–RNA interactions should be validated using orthogonal approaches (e.g., RIP–seq, CLIP–seq, or targeted pulldown assays), and mechanistic claims should be examined in established HGSOC models and, where possible, in patient–derived systems.

## 4. Materials and Methods

### 4.1. MNX1–AS1 Structure Prediction

For long non–coding secondary structure, the RNAFold web server [29,30] was used to translate base pair sequence into dot and bracket notation. Finally, to obtain the 3D structure of *MNX1*–*AS1*, the dot and bracket notation was used in the 3dRNA/DNA web server [31], obtaining the final structure.

### 4.2. Bioinformatic Data Collection and Preprocessing

The analysis was conducted using RIblast v1.2 [32], a computational tool designed for comparative genomics and evolutionary studies (with the following flags: RepeatFlag: 0, MaximalSpan: 70, MinAccessibleLength: 5, MaxSeedLength: 20, InteractionEnergyThreshold: −4, HybridEnergyThreshold: −6, FinalThreshold:−8, DropOutLengthWoGap: 5, DropOutLengthWGap: 16). In RIblast, hybridization energy refers to the predicted change in free energy (ΔG, typically reported in kcal/mol) when two RNA molecules form base–paired interactions to create an RNA–RNA duplex. It reflects the thermodynamic favorability of the interaction: more negative ΔG values indicate a more stable and energetically favorable pairing, whereas values closer to zero indicate weaker or unlikely hybridization. RIblast estimates this value using standard RNA thermodynamic parameters for base pairing and stacking, and we used it as a quantitative criterion to rank and filter predicted RNA–RNA interactions.

The analysis was performed in 3 steps: (1) general miRNA, (2) general mRNA, and (3) EMT–WNT mRNA analysis. To obtain the list of every mRNA relevant for this research, the molecular signature of the GSEA database [33] was used, as it keeps an actualized and curated list of genes involved in several biological processes. To achieve this point, either the computational gene sets or C4 were used. The C4 collection is divided into three subcollections: 3CA, CGN and CM, which have 149, 427 and 431 genes set, respectively (a gene set is a combination of several genes that belong specifically to a category, biological process or hallmark). For a correct download of the datasets, each gene present in the C4 database was downloaded using the NCBI Datasets software. Sequences were preprocessed to remove any redundant or incomplete entries, ensuring the quality and integrity of the dataset, keeping just the most expressed mRNA from each gene. In the case of miRNA, the database is shorter than the mRNA database, so every sequence that belongs to miRNA was downloaded and filtered following the same conditions as before. Finally, for step (1), 2130 gene sequences were used, for step (2), 5656 gene sequences were used, and for step (3), 344 gene sequences were used.

### 4.3. RIblast Analysis and Statistics

RIblast analysis was performed using default parameters unless otherwise specified. Briefly, RIblast employs a rapid indexing algorithm to efficiently identify homologous regions between genomic sequences. The algorithm utilizes a combination of k–mer indexing and seed–and–extend approaches to detect conserved regions, allowing for the identification of evolutionary relationships among sequences. So, for each analysis step, the long non–coding sequences selected as targets belong to *MNX1*–*AS1*. To prevent bias between sequences, analysis was performed in both T > U and T < U sequences to identify some variations between the base pair content. Nevertheless, the results for both analyses were the same. Statistical analyses, including bootstrap resampling and branch support estimation, were performed to assess the robustness and reliability of the results obtained. The results were normalized by z–score due the robustness of this method, especially in identification and normalization of differences in sequence length. To identify the best interacting candidate, the interaction energy was normalized by the length of the detected interacting sequence, and candidates were then ranked and selected according to quartile divisions.

### 4.4. RNAseq Analysis and Statistics

To undercover *MNX1*–*AS1*–associated gene expression, an RNAseq analysis was performed. RNAseq data for gene expression analysis were obtained from The Cancer Genome Atlas (TCGA) for ovarian cancer samples (488 samples = from the project IDs of TCGA–OV and CPTAC–2) and from the Genotype–Tissue Expression (GTEx) project for normal tissue controls (193 samples = from Bulk Tissue Expression, Analysis V8, version 1.1.9). Cancer patients were stratified into four groups based on cancer stages (I, II, III, and IV). A second RNAseq analysis was performed, according to our previous data published, from parental A2780 and CBDCA–resistant A2780 (CBDCA–R–A2780) [34] cell lines following these steps: (i) Adapter trimming was performed with the software Trimmomatic v0.36 [35] (with the following flags: trimmomatic PE –phred33 LEADING:3 TRAILING:3 SLIDINGWINDOW:4:15 MINLEN:36 HEADCROP:15 ILLUMINACLIP:TruSeq3–PE.fa:2:30:10). (ii) Ribosomal RNA (rRNA) was filtered from samples utilizing sortmerna v4.3.7 [36] (with the following flags: –ref silva–bac–23s–id98.fasta –ref silva–arc–16s–id95.fasta –ref silva–arc–23s–id98.fasta –ref silva–euk–18s–id95.fasta –ref silva–euk–28s–id98.fasta –ref rfam–5s–database–id98.fasta –ref rfam–5.8s–database–id98.fasta —sam —num_alignments 1 —fastx —paired_in —out2). (iii) STAR v2.7.11b [37] was used for Mapping the samples against references genome Hg38 v113—ENSEMBL (with the following flags: —outSAMtype BAM SortedByCoordinate —outSAMunmapped Within –outSAMattributes Standard —twopassMode Basic —outSAMmultNmax 1 —outFileNamePrefix Alignment). (iv) Counting was performed with featureCounts v2.0.0 [38] (with the following flags: –p –O —primary –t exon).

The analysis of these samples was performed following the same methodology as the TCGA–OV/GTEx samples. Bioinformatics analysis relied solely on read counts obtained from RNAseq data. Differential gene expression between cancer and normal tissue datasets were assessed using DESeq2 v.1.36.0 [39] in R (4.2.1), with genes showing a log2 fold change ≥ 1 and a *p*-value < 0.05 considered significantly differentially expressed. For correlation analysis, raw counts were transformed utilizing the variance–stabilizing transformation (VST) formula in order to minimize any dependence on the variability. Ontology enrichment analysis was performed using the library enrichGO [40] with the following flags: pAdjustMethod = “BH”, pvalueCutoff = 0.05, minGSSize = 5, maxGSSize = 3000.

### 4.5. Spearman Correlation

The Spearman correlation coefficient was computed using normalized raw count data. Genes were then stratified into positively and negatively correlated subsets based on the distribution of correlation coefficients, with thresholds defined by quantile separation. In order to identify and filter TCGA DEGs, correlated TCGA genes were filtered by correlated A2780 genes following these steps: (i) calculation of Spearman’s correlation between MNX1–AS1 in TCGA samples, (ii) calculation of Spearman’s correlation between MNX1–AS1 in resistant cell lines A2780 UCI, (iii) filtering of TCGA Spearman–correlated genes by resistant cell lines A2780 UCI.

### 4.6. Plotting and Visualization

For visualization and plotting, R (4.2.1) and Rstudio 2024.12.0+467 were utilized.

## 5. Conclusions

Our study identifies MNX1–AS1 as a strongly upregulated lncRNA in the carboplatin–resistant A2780 model and shows that it is also elevated in TCGA ovarian tumor datasets. Together, these observations prioritize MNX1–AS1 as a candidate associated with resistance–like transcriptional features; however, concordant expression does not establish mechanistic equivalence between A2780 and HGSOC or imply shared global resistance programs.

Our RNA–RNA interaction predictions and transcriptomic integration highlight a subset of EMT–related transcripts (including SNAI2, FOXA1, and ZEB1) that are both upregulated and predicted to interact with MNX1–AS1. These in silico predictions provide hypotheses for future testing rather than definitive evidence of direct regulation.

Overall, MNX1–AS1 remains a promising prognostic biomarker and a potential therapeutic target candidate, but mechanistic and subtype–general conclusions will require validation in established HGSOC models and/or patient–derived systems using functional perturbation and orthogonal interaction assays.

## Data Availability

The raw data supporting the conclusions of this article will be made available by the authors on request.

## Supplementary Materials

The following supporting information can be downloaded at: https://www.mdpi.com/article/10.3390/ijms27083428/s1.

## Abbreviations

The following abbreviations are used in this manuscript:

OC	Ovarian Cancer
lncRNAs	Long Non–Coding RNA
EMT	Epithelial–Mesenchymal Transition
miRNA	Micro RNA
mRNA	Messenger RNA
EOC	Epithelial Ovarian Cancer
HGSOC	High–Grade Serous Ovarian Cancer
TCGA	The Cancer Genome Atlas
DEG	Differentially Expressed Gene
GTEx	Genotype–Tissue Expression
NCBI	National Center for Biotechnology Information
OS	Overall Survival
PFS	Progression–Free Survival
ceRNA	Competing Endogenous RNA
CBDCA	Carbodicloacetate
FIGO	Fédération Internationale de Gynécologie et d’Obstétrique
RIP–seq	RNA Immunoprecipitation Sequencing
GSEA	Gene Set Enrichment Analysis

## References

[1] Siegel R.L., Miller K.D., Fuchs H.E., Jemal A. (2021). Cancer Statistics, 2021. CA Cancer J. Clin..

[2] Jayson G.C., Kohn E.C., Kitchener H.C., Ledermann J.A. (2014). Ovarian cancer. Lancet.

[3] Lheureux S., Gourley C., Vergote I., Oza A.M. (2019). Epithelial ovarian cancer. Lancet.

[4] Chen W., Zheng R., Baade P.D., Zhang S., Zeng H., Bray F., Jemal A., Yu X.Q., He J. (2016). Cancer statistics in China, 2015. CA Cancer J. Clin..

[5] Konstantinopoulos P.A., Norquist B., Lacchetti C., Armstrong D., Grisham R.N., Goodfellow P.J., Kohn E.C., Levine D.A., Liu J.F., Lu K.H. (2020). Germline and Somatic Tumor Testing in Epithelial Ovarian Cancer: ASCO Guideline. J. Clin. Oncol..

[6] Cannistra S.A. (2004). Cancer of the Ovary. N. Engl. J. Med..

[7] du Bois A., Lück H.J., Meier W., Adams H.P., Möbus V., Costa S., Bauknecht T., Richter B., Warm M., Schröder W. (2003). A Randomized Clinical Trial of Cisplatin/Paclitaxel Versus Carboplatin/Paclitaxel as First–Line Treatment of Ovarian Cancer. J. Natl. Cancer Inst..

[8] Hoogstraat M., de Pagter M.S., Cirkel G.A., van Roosmalen M.J., Harkins T.T., Duran K., Kreeftmeijer J., Renkens I., Witteveen P.O., Lee C.C. (2014). Genomic and transcriptomic plasticity in treatment–naïve ovarian cancer. Genome Res..

[9] Guttman M., Amit I., Garber M., French C., Lin M.F., Feldser D., Huarte M., Zuk O., Carey B.W., Cassady J.P. (2009). Chromatin signature reveals over a thousand highly conserved large non–coding RNAs in mammals. Nature.

[10] Clark M.B., Mercer T.R., Bussotti G., Leonardi T., Haynes K.R., Crawford J., Brunck M.E., Le Cao K.-A., Thomas G.P., Chen W.Y. (2015). Quantitative gene profiling of long noncoding RNAs with targeted RNA sequencing. Nat. Methods.

[11] Sun M., Kraus W.L. (2015). From Discovery to Function: The Expanding Roles of Long NonCoding RNAs in Physiology and Disease. Endocr. Rev..

[12] Quinn J.J., Chang H.Y. (2016). Unique features of long non–coding RNA biogenesis and function. Nat. Rev. Genet..

[13] Flynn R.A., Chang H.Y. (2014). Long Noncoding RNAs in Cell–Fate Programming and Reprogramming. Cell Stem Cell.

[14] Mercer T.R., Mattick J.S. (2013). Structure and function of long noncoding RNAs in epigenetic regulation. Nat. Struct. Mol. Biol..

[15] Wang K.C., Chang H.Y. (2011). Molecular Mechanisms of Long Noncoding RNAs. Mol. Cell.

[16] Schmitt A.M., Chang H.Y. (2016). Long Noncoding RNAs in Cancer Pathways. Cancer Cell.

[17] Meryet-Figuière M., Lambert B., Gauduchon P., Vigneron N., Brotin E., Poulain L., Denoyelle C. (2016). An overview of long non–coding RNAs in ovarian cancers. Oncotarget.

[18] Qiu J.-J., Lin Y.-Y., Ye L.-C., Ding J.-X., Feng W.-W., Jin H.-Y., Zhang Y., Li Q., Hua K.-Q. (2014). Overexpression of long non–coding RNA HOTAIR predicts poor patient prognosis and promotes tumor metastasis in epithelial ovarian cancer. Gynecol. Oncol..

[19] Zhou Y., Xu X., Lv H., Wen Q., Li J., Tan L., Li J., Sheng X. (2016). The Long Noncoding RNA MALAT–1 Is Highly Expressed in Ovarian Cancer and Induces Cell Growth and Migration. PLoS ONE.

[20] Jin Y., Feng S.-J., Qiu S., Shao N., Zheng J.-H. (2017). LncRNA MALAT1 promotes proliferation and metastasis in epithelial ovarian cancer via the PI3K–AKT pathway. Eur. Rev. Med. Pharmacol. Sci..

[21] Abildgaard C., Canto L.M.D., Steffensen K.D., Rogatto S.R. (2020). Long Non–coding RNAs Involved in Resistance to Chemotherapy in Ovarian Cancer. Front. Oncol..

[22] Chen Y., Bi F., An Y., Yang Q. (2019). Identification of pathological grade and prognosis–associated lncRNA for ovarian cancer. J. Cell. Biochem..

[23] Li M., Yan Y., Liu Y., Zhao J., Guo F., Chen J., Nie L., Zhang Y., Wang Y. (2023). Comprehensive analyses of fatty acid metabolism–related lncRNA for ovarian cancer patients. Sci. Rep..

[24] Zhang X., Zhang Y., Liu Q., Zeng A., Song L. (2024). Glycolysis–associated lncRNAs in cancer energy metabolism and immune microenvironment: A magic key. Front. Immunol..

[25] Fang L., Wang H., Li P. (2018). Systematic analysis reveals a lncRNA–mRNA co–expression network associated with platinum resistance in high–grade serous ovarian cancer. Investig. New Drugs.

[26] Xu J., Wu J., Fu C., Teng F., Liu S., Dai C., Shen R., Jia X. (2018). Multidrug resistant lncRNA profile in chemotherapeutic sensitive and resistant ovarian cancer cells. J. Cell. Physiol..

[27] Li T., Zhou S., Yang Y., Xu Y., Gong X., Cheng Y., Wang Y. (2022). LncRNA MNX1–AS1: A novel oncogenic propellant in cancers. Biomed. Pharmacother..

[28] Shuai Y., Ma Z., Yue J., Li C., Ju J., Wang X., Qian H., Yuan P. (2025). MNX1–AS1 suppresses chemosensitivity by activating the PI3K/AKT pathway in breast cancer. Int. J. Biol. Sci..

[29] Hofacker I.L., Fontana W., Stadler P.F., Bonhoeffer L.S., Tacker M., Schuster P. (1994). Fast folding and comparison of RNA secondary structures. Monatsh. Chem. Chem. Mon..

[30] Lorenz R., Bernhart S.H., Honer Zu Siederdissen C., Tafer H., Flamm C., Stadler P.F., Hofacker I.L. (2011). ViennaRNA Package 2.0. Algorithms Mol. Biol..

[31] Zhang Y., Xiong Y., Yang C., Xiao Y. (2024). 3dRNA/DNA: 3D Structure Prediction from RNA to DNA. J. Mol. Biol..

[32] Fukunaga T., Hamada M. (2017). RIblast: An ultrafast RNA–RNA interaction prediction system based on a seed–and–extension approach. Bioinformatics.

[33] Subramanian A., Tamayo P., Mootha V.K., Mukherjee S., Ebert B.L., Gillette M.A., Paulovich A., Pomeroy S.L., Golub T.R., Lander E.S. (2005). Gene set enrichment analysis: A knowledge–based approach for interpreting genome–wide expression profiles. Proc. Natl. Acad. Sci. USA.

[34] Viscarra T., Buchegger K., Jofre I., Riquelme I., Zanella L., Abanto M., Parker A.C., Piccolo S.R., Roa J.C., Ili C. (2019). Functional and transcriptomic characterization of carboplatin–resistant A2780 ovarian cancer cell line. Biol. Res..

[35] Bolger A.M., Lohse M., Usadel B. (2014). Trimmomatic: A flexible trimmer for Illumina sequence data. Bioinformatics.

[36] Kopylova E., Noé L., Touzet H. (2012). SortMeRNA: Fast and accurate filtering of ribosomal RNAs in metatranscriptomic data. Bioinformatics.

[37] Dobin A., Davis C.A., Schlesinger F., Drenkow J., Zaleski C., Jha S., Batut P., Chaisson M., Gingeras T.R. (2013). STAR: Ultrafast universal RNA–seq aligner. Bioinformatics.

[38] Liao Y., Smyth G.K., Shi W. (2014). featureCounts: An efficient general purpose program for assigning sequence reads to genomic features. Bioinformatics.

[39] Love M.I., Huber W., Anders S. (2014). Moderated estimation of fold change and dispersion for RNA–seq data with DESeq2. Genome Biol..

[40] Yu G., Wang L.-G., Han Y., He Q.-Y. (2012). clusterProfiler: An R Package for Comparing Biological Themes Among Gene Clusters. OMICS J. Integr. Biol..

